# Gene expression of bacterial collagenolytic proteases in root caries

**DOI:** 10.1080/20002297.2018.1424475

**Published:** 2018-01-18

**Authors:** Nailê Damé-Teixeira, Clarissa Cavalcanti Fatturi Parolo, Marisa Maltz, Ariel Goulart Rup, Deirdre Ann Devine, Thuy Do

**Affiliations:** ^a^ Faculty of Health Science, Department of Dentistry, University of Brasilia, Brasilia, Brazil; ^b^ Faculty of Dentistry, Department of Social and Preventive Dentistry, Federal University of Rio Grande do Sul, Porto Alegre, Brazil; ^c^ School of Dentistry, Division of Oral Biology, University of Leeds, Leeds, United Kingdom

**Keywords:** Root caries, gene expression, collagen, microbial collagenase, sequence analysis, RNA, biofilms

## Abstract

**Objective:** It is unknown whether bacteria play a role in the collagen matrix degradation that occurs during caries progression. Our aim was to characterize the expression level of genes involved in bacterial collagenolytic proteases in root biofilms with and without caries. **Method:** we collected samples from active cavitated root caries lesions (RC, n = 30) and from sound root surfaces (SRS, n = 10). Total microbial RNA was isolated and cDNA sequenced on the Illumina Hi-Seq2500. Reads were mapped to 162 oral bacterial reference genomes. Genes encoding putative bacterial collagenolytic proteases were identified. Normalization and differential expression analysis was performed on all metatranscriptomes (FDR<10-3).

**Result:** Genes encoding collagenases were identified in 113 bacterial species the majority were peptidase U32. In RC, Streptococcus mutans and Veillonella parvula expressed the most collagenases. Organisms that overexpressed collagenolytic protease genes in RC (Log2FoldChange>8) but none in SRS were Pseudoramibacter alactolyticus [HMPREF0721_RS02020; HMPREF0721_RS04640], Scardovia inopinata [SCIP_RS02440] and Olsenella uli DSM7084 [OLSU_RS02990].

**Conclusion:** Our findings suggest that the U32 proteases may be related to carious dentine. The contribution of a small number of species to dentine degradation should be further investigated. These proteases may have potential in future biotechnological and medical applications, serving as targets for the development of therapeutic agents.

## Introduction

Root hard tissues (cementum and dentine) become vulnerable to demineralization once root surfaces are exposed. These tissues are less mineralized than enamel and are composed of high proportions of organic materials such as collagen [1,2]. From a clinical point of view, the development of caries in root hard tissues may be considered a two-stage process: the first stage is characterized by mineral dissolution and the second by the degradation of the organic matrix of the root surface [3]. Microbial invasion of cementum and dentine tissues has been reported even in the first stage of the caries process, whereas in enamel caries, dentine is invaded only once enamel is destroyed [4,5]. This fact has an impact on the microbiome associated with the caries process in root hard tissues.

The function of bacteria in the demineralization stage of caries development is well known. Root hard tissue demineralization may develop in the presence of a rich and diverse microbiota, and the acidification of the microenvironment selects some species that are able to survive at low pH and produce high amounts of organic acids [6]. Root dentine biofilms are composed of a variety of saccharolytic, aciduric, and acidogenic organisms, as well as proteolytic bacteria, which can produce acids or ammonia from the catabolism of nitrogenous substrates that are available exogenously or from the dentine organic matrix [3,7]; thus, they can affect the biofilm pH in several ways. In addition to demineralization, bacteria may be involved in matrix degradation. Collagen is resistant to most common proteases and can be degraded by only a few types of proteases from mammals or bacteria [8], including some metalloproteases and serine proteases. It has been suggested that host collagenases from dentine are associated with collagen matrix degradation during caries progression [9,10], representing a response of the host tissues to caries attack under acidic conditions. These proteases, which include matrix metalloproteinases (MMP-2, 3, 8, 9, and 20) and cysteine cathepsins (B and K), are present in the dentinal organic matrix and become activated once the cementum is degraded [3,9–13].

Recently, a tissue-dependent hypothesis for dental caries suggested that some bacteria could promote dentine degradation and caries development [14]. This hypothesis is based on the discovery of overexpression of genes related to proteolytic activity, as well as bacterial collagenases in dentinal caries from coronal lesions [14,15]. These studies showed for the first time that microbial proteolytic activity might contribute to dentinal protein degradation. Microbial collagenolytic activity has been demonstrated in a few oral bacteria [3]; however, a real contribution of bacteria to the degradation of the organic part of root dentine remains questionable. Protease PrtC from *Porphyromonas gingivalis* ATCC 53977 is one of the most reported microbial collagenolytic proteases produced by oral bacteria. It is part of the U32 protease family and contains 1,002 bp encoding a 333-residue PrtC protein. It can degrade soluble and reconstituted fibrillar type I collagen (the most common in root hard tissues) at body temperature or below [8,16]. Due to the relationship between the periodontal biofilm and the biofilm that cause root caries (RC), this protein could be involved in root dentine degradation.

The collagenase activity-dependent ability to degrade the dentinal collagen matrix could be an important virulence trait of plaque biofilms. In this study, we evaluated bacterial collagenolytic protease gene expression within natural biofilms from RC compared with supragingival biofilms of RC-free individuals by RNA-seq data analysis. The terminology ‘bacterial collagenolytic proteases’ was used to refer to all proteases that can degrade at least one type of collagen according to Zhang et al., including true collagenases and other proteases with collagenolytic activity [8]. These data may help clarify the role of bacteria in collagen matrix degradation in RC.

## Materials and methods

Sample collection was carried out as described by Damé-Teixeira et al. [17]. Briefly, 10 volunteers with an exposed root surface and no RC lesion were included in the sound root surface group (SRS). Supragingival biofilms were collected from all exposed root surfaces. All participants recruited for the RC group had one primary cavitated root lesion in need of restorative treatment. All lesions presented characteristics of activity (soft and yellow dentine). Biofilm and carious dentine samples (soft and infected tissue) were collected from 30 patients during the restorative treatment.

Upon collection, samples were placed in a nuclease-free microtube containing 1 mL of RNAprotect reagent (Qiagen Inc.). Total RNA was extracted from all samples using the UltraClean® Microbial RNA Isolation (Mo-bio, San Diego, CA, USA) using on-column DNAse digestion (Qiagen, Inc.). The extracted RNA was quantified using the Quant-iT™ RiboGreen® RNA Assay Kit (Invitrogen), and samples with total RNA concentration <30 ng/RNA were pooled, leading to a final sample count of 10 SRS and 9 RC. The Ribo-Zero™ Meta-Bacteria Kit (Epicentre, Illumina) was used for mRNA enrichment, and Illumina®TruSeq™ library prep protocols (Illumina, SD) were used for library preparation and paired-end sequencing with the Illumina HiSeq2500.

Read sequences for each sample were quality trimmed using cutadapt and imported into the CLC Genomics Workbench v8 software (CLC Bio, Qiagen). The genomes of 162 bacteria and their associated information were downloaded from the DNA Data Bank of Japan, NCBI, the Broad Institute, and the HOMD database and mapped against the short-reads sequences (for the list of genomes, see [17]). The data produced are available from the National Center for Biotechnology Information (NCBI) Sequence Read Archive, under the accession numbers SRS779973 and SRS796739. Read count data for all potential collagenases were manually extracted from the 162 genomes, with particular focus on the U32 family proteases [8] due to the implication of this family as virulence factors in oral bacteria and its abundance. However, peptidolytic or gelatinolytic proteases were not included in this study’s analysis.

The number of genes with no activity was stated as ‘number of reads = 0’. The relative median expression level for genes from bacterial collagenolytic proteases was calculated for each of the sample groups, as described previously [18] within the R package ‘DESeq’ [19], and considered as the ‘gene expression value’. Graphs were generated within the R package ‘plotly’ [20].

Statistical analysis for inferring differential gene expression between sample groups was also carried out using the R package DESeq2 [21]. The cut-off for designating a gene as being differentially expressed was a change in transcript levels of at least 1-log fold change (two times difference, negative values = up-regulated in SRS and down-regulated in RC and positive values = down-regulated in RC and up-regulated in SRS) and Benjamini–Hochberg adjusted *p*-value (padj) of less than 10^−^^3^ [22]. This high cut-off was chosen in order to avoid false-positive results and identify only true differences.

This study was approved by the ethics committee of the Federal University of Rio Grande do Sul (process n° 427.168) and by the Yorkshire & The Humber – Leeds West National Research Ethics Service Committee (protocol n° 2012002DD). Volunteers to the study were patients who attended dental clinics for any dental treatment in two centres: Faculty of Dentistry, Federal University of Rio Grande do Sul, Porto Alegre, Brazil; and the School of Dentistry, Dental Translational Research Unit, University of Leeds, Leeds, UK. All volunteers consented to participate and donate samples after receiving the information about the study.

## Results

A total of 201 genes coding for bacterial collagenolytic proteases were identified in 113 bacterial species; 24 from *Prevotella* spp. and 20 from *Streptococcus* spp. Table 1 describes genes encoding bacterial collagenolytic proteases identified in the metatranscriptome analysis of root biofilms, showing that a majority expressed genes for the peptidase U32 family (basically protease PrtC).

**Table 1.** List of genes coding for bacterial collagenolytic proteases.

Genome	Locus tag	Protein product	Protein annotation
*Aggregatibacter actinomycetemcomitans* D11S-1	D11S_1802	ACX83163.1	Peptidase U32
*Aggregatibacter aphrophilus* NJ8700 [23]	NT05HA_RS01075	WP_005701395.1	Collagenase-like protease, PrtC family
*Alloprevotella tannerae* ATCC 51259	GCWU000325_RS05925	WP_006255366.1	Collagenase
*Alloprevotella tannerae* ATCC 51259	GCWU000325_RS08705	WP_006256078.1	Collagenase-like protease, PrtC family
*Alloprevotella tannerae* ATCC 51259	GCWU000325_RS05925	WP_006255366.1	Collagenase
*Atopobium rimae* ATCC 49626	ATORI0001_RS02210	WP_003148443.1	Peptidase U32
*Bifidobacterium breve* UCC2003 [24]	BBR_RS19280	WP_015439232.1	Peptidase U32
*Bifidobacterium dentium* Bd1	BDP_RS01815	WP_012901869.1	Collagenase
*Bifidobacterium kashiwanohense* PV20-2	AH68_RS01480	WP_039196994.1	Collagenase
*Bifidobacterium thermophilum* RBL67	D805_RS06990	WP_044282489.1	Collagenase
*Campylobacter concisus* 13826	CCC13826_RS05820	WP_048809830.1	Collagenase-like protease, PrtC family
*Campylobacter curvus* 525.92	CCV52592_RS06400	WP_011992484.1	Collagenase-like protease, PrtC family
*Campylobacter gracilis* strain ATCC 33236	CGRAC_RS08900	WP_005873169.1	Collagenase-like protease, PrtC family
*Campylobacter rectus* RM3267	CAMRE0001_RS04590	WP_004318907.1	Collagenase-like protease, PrtC family
Candidate division SR1 bacterium Aalborg_AAW-1	XF24_00476	AKH32809.1	Collagenase
*Capnocytophaga gingivalis* ATCC 33624	CAPGI0001_RS09340	WP_002669179.1	Collagenase
*Capnocytophaga sputigena* ATCC 33612	CAPSP0001_RS09700	WP_002680903.1	Collagenase
*Cardiobacterium hominis* ATCC 15826	HMPREF0198_RS02295	WP_004139642.1	Collagenase-like protease, PrtC family
*Catonella morbi* ATCC 51271	GCWU000282_RS01655	WP_035039351.1	Peptidase U32
*Catonella morbi* ATCC 51271	GCWU000282_RS01780	WP_023353272.1	Peptidase U32
*Clostridium saccharolyticum* WM1WM1	CLOSA_RS11275	WP_013272899.1	Peptidase U32
*Clostridium saccharolyticum* WM1WM1	CLOSA_RS04645	WP_013271613.1	Peptidase U32
*Dialister invisus* DSM 15470	GCWU000321_RS07150	WP_007070508.1	Peptidase U32
*Dichelobacter nodosus* VCS1703A	DNO_RS01910	WP_012030735.1	Collagenase-like protease, PrtC family
*Eikenella corrodens* ATCC 23834	EIKCOROL_RS00310	WP_003821765.1	Collagenase-like protease, PrtC family
*Eikenella corrodens* ATCC 23834	EIKCOROL_RS00745	WP_035579870.1	Collagenase-like protease, PrtC family
*Eubacterium eligens* ATCC 27750	EUBELI_RS04560	WP_012739182.1	Collagenase-like protease, PrtC family
*Eubacterium eligens* ATCC 27750	EUBELI_RS03735	WP_041688528.1	Peptidase U32
*Eubacterium saphenum* ATCC 49989	GCWU000322_RS00740	WP_005837827.1	Peptidase U32
*Filifactor alocis* ATCC 35896	HMPREF0389_RS02570	WP_014262170.1	Protease
*Filifactor alocis* ATCC 35896	HMPREF0389_RS00640	WP_014261808.1	Peptidase U32
*Fusobacterium nucleatum* subsp. *animalis* 7_1	FSDG_RS09345	WP_008702184.1	Collagenase-like protease, PrtC family
*Fusobacterium nucleatum* subsp. *animalis* 7_1	FSDG_RS08285	WP_008702419.1	Collagenase-like protease, PrtC family
*Fusobacterium nucleatum* subsp. *nucleatum* ATCC 25586	FN1931	NP_602731.1	Collagenase-like protease, PrtC family
*Fusobacterium nucleatum* subsp. *nucleatum* ATCC 25586	FN1826	NP_602626.1	Collagenase-like protease, PrtC family
*Fusobacterium nucleatum* subsp. *vincentii* 3_1_36A2	HMPREF0946_RS00995	WP_008800359.1	Collagenase-like protease, PrtC family
*Fusobacterium nucleatum* subsp. *vincentii* 3_1_36A2	HMPREF0946_RS02055	WP_008796619.1	Collagenase-like protease, PrtC family
*Fusobacterium periodonticum* ATCC 33693	FUSPEROL_RS01940	WP_039984117.1	Collagenase-like protease, PrtC family
*Fusobacterium periodonticum* ATCC 33693	FUSPEROL_RS01460	WP_005970981.1	Collagenase-like protease, PrtC family
*Gemella haemolysans* M341	HMPREF0428_RS03755	WP_003146785.1	Collagenase-like protease, PrtC family
*Gemella haemolysans* M341	HMPREF0428_RS03760	WP_003146787.1	Peptidase U32
*Gemella moribillum* M424	HMPREF0432_RS03545	WP_004632787.1	Collagenase-like protease, PrtC family
*Gemella moribillum* M424	HMPREF0432_RS03550	WP_004632788.1	Peptidase U32
*Granulicatella adiacens* ATCC 49175	HMPREF0444_RS08360	WP_005607759.1	Collagenase-like protease, PrtC family
*Granulicatella adiacens* ATCC 49175	HMPREF0444_RS08365	WP_005607760.1	Peptidase U32
*Granulicatella elegans* ATCC 700633	HMPREF0446_RS01120	WP_006703604.1	Peptidase U32
*Granulicatella elegans* ATCC 700633	HMPREF0446_RS01125	WP_006703603.1	Collagenase-like protease, PrtC family
*Haemophilus influenzae* F3047	HICON_RS03890	WP_013526492.1	Collagenase-like protease, PrtC family
*Haemophilus parainfluenzae* T3T1	PARA_RS00685	WP_005695474.1	Collagenase-like protease, PrtC family
*Kingella oralis* ATCC 51147	GCWU000324_RS07250	WP_003796734.1	Collagenase-like protease, PrtC family
*Lachnoanaerobaculum saburreum* DSM 3986	HMPREF0381_RS09405	WP_040461351.1	Peptidase U32
*Lachnoanaerobaculum saburreum* DSM 3986	HMPREF0381_RS11935	WP_008752244.1	Peptidase U32
*Lautropia mirabilis*	HMPREF0551_RS01810	WP_040529625.1	Collagenase-like protease, PrtC family
*Leptotrichia buccalis* [25]	LEBU_RS05040	WP_041760865.1	Peptidase U32
*Leptotrichia buccalis* [25]	LEBU_RS10190	WP_015770252.1	Collagenase-like protease, PrtC family
*Neisseria bacilliformis*	HMPREF9123_RS07480	WP_007342950.1	Collagenase-like protease, PrtC family
*Neisseria bacilliformis*	HMPREF9123_RS08480	WP_007343222.1	Collagenase-like protease, PrtC family
*Neisseria elongata* subsp. *glycolytica* ATCC 29315 [26]	NELON_RS10680	WP_003769563.1	Collagenase-like protease, PrtC family
*Neisseria elongata* subsp. *glycolytica* ATCC 29315 [26]	NELON_RS07015	WP_003771571.1	Collagenase-like protease, PrtC family
*Neisseria flavescens* SK114	NEIFL0001_RS03385	WP_003684307.1	Collagenase-like protease, PrtC family
*Neisseria flavescens* SK114	NEIFL0001_RS01600	WP_003683417.1	Collagenase-like protease, PrtC family
*Neisseria lactamica* 020-06 [27]	NLA_RS03260	WP_013448613.1	Collagenase-like protease, PrtC family
*Neisseria mucosa* C102	HMPREF0604_RS08420	WP_003748766.1	Collagenase-like protease, PrtC family
*Neisseria mucosa* C102	HMPREF0604_RS07970	WP_003748589.1	Collagenase-like protease, PrtC family
*Neisseria subflava*	NEISUBOT_RS03855	WP_004519592.1	Collagenase-like protease, PrtC family
*Neisseria subflava*	NEISUBOT_RS04305	WP_004519683.1	Collagenase-like protease, PrtC family
*Olsenella uli* DSM7084 [28]	OLSU_RS02990	WP_041549197.1	Peptidase U32
*Oribacterium* sp. oral taxon 078 str. F0262	GCWU000341_RS02740	WP_009214193.1	Peptidase U32
*Parvimonas micra* ATCC 33270	PEPMIC_RS07615	WP_004833569.1	Peptidase U32
*Parvimonas micra* ATCC 33270	PEPMIC_RS08350	WP_041954953.1	Peptidase U32
*Peptostreptococcus stomatis*	HMPREF0634_RS06810	WP_007790248.1	Collagenase-like protease, PrtC family
*Peptostreptococcus stomatis*	HMPREF0634_RS00830	WP_007788149.1	Peptidase U32
*Porphyromonas asaccharolytica* DSM 20707	PORAS_RS01075	WP_013759854.1	Peptidase U32
*Porphyromonas asaccharolytica* DSM 20707	PORAS_RS04355	WP_013760316.1	Collagenase
*Porphyromonas endodontalis*	POREN0001_RS06205	WP_004334244.1	Peptidase U32
*Porphyromonas endodontalis*	POREN0001_RS08830	WP_052296722.1	Collagenase
*Porphyromonas gingivalis* ATCC 33277 [29]	PGN_RS02685	WP_039416961.1	Collagenase
*Porphyromonas gingivalis* ATCC 33277 [29]	PGN_RS03720	WP_012457772.1	Collagenase
*Porphyromonas gingivalis* ATCC 33277 [29]	PGN_RS02685	WP_039416961.1	Collagenase
*Porphyromonas gingivalis* ATCC 33277 [29]	PGN_RS03720	WP_012457772.1	Collagenase
*Prevotella amnii* DSM 23384 = JCM 14753	F596_RS0106960	WP_026302377.1	Collagenase-like protease, PrtC family
*Prevotella amnii* DSM 23384 = JCM 14753	F596_RS0104625	WP_019036032.1	Collagenase
*Prevotella bergensis*	HMPREF0645_RS06070	WP_007174036.1	Collagenase-like protease, PrtC family
*Prevotella bergensis*	HMPREF0645_RS09130	WP_007174691.1	Collagenase
*Prevotella bivia* DSM 20514	PREBIDRAFT_RS05630	WP_004336717.1	Collagenase
*Prevotella bryantii* C21a	G638_RS0101200	WP_027452885.1	Collagenase-like protease, PrtC family
*Prevotella bryantii* C21a	G638_RS0104370	WP_027453233.1	Collagenase
*Prevotella buccae* ATCC 33574	HMPREF6485_RS08010	WP_044125959.1	Collagenase-like protease, PrtC family
*Prevotella buccae* ATCC 33574	HMPREF6485_RS03410	WP_004341966.1	Collagenase
*Prevotella buccalis* ATCC 35310	HMPREF0650_RS10745	WP_004350712.1	Collagenase-like protease, PrtC family
*Prevotella buccalis* ATCC 35310	HMPREF0650_RS05375	WP_004348964.1	Collagenase
*Prevotella copri*	PREVCOP_RS02145	WP_006846714.1	Collagenase-like protease, PrtC family
*Prevotella copri*	PREVCOP_RS08960	WP_006848256.1	Collagenase
*Prevotella dentalis* DSM 3688	PREDE_RS10275	WP_005847167.1	Collagenase-like protease, PrtC family
*Prevotella dentalis* DSM 3688	PREDE_RS07105	WP_005845431.1	Collagenase
*Prevotella denticola* F0289	HMPREF9137_RS07010	WP_013671465.1	Collagenase-like protease, PrtC family
*Prevotella denticola* F0289	HMPREF9137_RS02430	WP_013670722.1	Collagenase
*Prevotella disiens* JCM 6334 = ATCC 29426	HMPREF0653_RS10015	WP_021670023.1	Peptidase U32
*Prevotella disiens* JCM 6334 = ATCC 29426	HMPREF0653_RS06040	WP_021669257.1	Collagenase
*Prevotella intermedia* 17 chr1 and 2	PIN17_RS08560	WP_014709923.1	Collagenase-like protease, PrtC family
*Prevotella intermedia* 17 chr1 and 2	PIN17_RS04505	WP_014709153.1	Collagenase
*Prevotella marshii* DSM 16,973 = JCM 13450	HMPREF0658_RS00735	WP_006947833.1	Collagenase-like protease, PrtC family
*Prevotella marshii* DSM 16,973 = JCM 13450	HMPREF0658_RS07640	WP_006949885.1	Collagenase
*Prevotella melaninogenica* ATCC 25845	HMPREF0659_RS08000	WP_013265267.1	Collagenase-like protease, PrtC family
*Prevotella melaninogenica* ATCC 25845	HMPREF0659_RS07035	WP_044045939.1	Collagenase
*Prevotella multiformis*	HMPREF9141_RS08830	WP_007368357.1	Collagenase-like protease, PrtC family
*Prevotella multiformis*	HMPREF9141_RS06410	WP_007367797.1	Collagenase
*Prevotella nigrescens* ATCC 33563	HMPREF9419_RS03665	WP_004366273.1	Collagenase-like protease, PrtC family
*Prevotella nigrescens* ATCC 33563	HMPREF9419_RS06820	WP_004366953.1	Collagenase
*Prevotella oralis* ATCC 33269	HMPREF0663_RS01445	WP_004368289.1	Collagenase-like protease, PrtC family
*Prevotella oralis* ATCC 33269	HMPREF0663_RS04675	WP_004369102.1	Collagenase
*Prevotella oris* F0302	HMPREF0971_RS00090	WP_004370849.1	Collagenase-like protease, PrtC family
*Prevotella oris* F0302	HMPREF0971_RS12160	WP_004375331.1	Collagenase
*Prevotella pallens* ATCC 700821	HMPREF9144_RS05520	WP_040595396.1	Collagenase-like protease, PrtC family
*Prevotella pallens* ATCC 700821	HMPREF9144_RS01260	WP_006043979.1	Collagenase
*Prevotella ruminicola* 23	PRU_RS03090	WP_013063209.1	Collagenase-like protease, PrtC family
*Prevotella ruminicola* 23	PRU_RS14605	WP_013065324.1	Collagenase
*Prevotella salivae* DSM 15606	HMPREF9420_RS01830	WP_007133684.1	Collagenase-like protease, PrtC family
*Prevotella salivae* DSM 15606	HMPREF9420_RS07750	WP_044079174.1	Collagenase
*Prevotella* sp. oral taxon 299 str. F0039	HMPREF0669_RS01095	WP_009228902.1	Collagenase-like protease, PrtC family
*Prevotella* sp. oral taxon 299 str. F0039	HMPREF0669_RS08220	WP_009227931.1	Collagenase
*Prevotella* sp. oral taxon 472 str. F0295	HMPREF6745_RS04325	WP_009235927.1	Collagenase-like protease, PrtC family
*Prevotella* sp. oral taxon 472 str. F0295	HMPREF6745_RS08625	WP_009236867.1	Collagenase
*Prevotella timonensis* 4401737	BN35_RS03705	WP_025071979.1	Collagenase-like protease, PrtC family
*Prevotella timonensis* 4401737	BN35_RS06480	WP_028900923.1	Collagenase
*Prevotella veroralis* F0319	HMPREF0973_RS06810	WP_004383383.1	Collagenase-like protease, PrtC family
*Prevotella veroralis* F0319	HMPREF0973_RS05650	WP_039851216.1	Collagenase
*Pseudoramibacter alactolyticus*	HMPREF0721_RS02020	WP_050786939.1	Collagenase-like protease, PrtC family
*Pseudoramibacter alactolyticus*	HMPREF0721_RS04640	WP_006598435.1	Peptidase U32
*Pyramidobacter piscolens*	HMPREF7215_RS04740	WP_040550474.1	Peptidase U32
*Scardovia inopinata* JCM 12537 [30]	SCIP_RS02440	WP_006292938.1	Peptidase U32
*Selenomonas infelix* ATCC 43532	HMPREF9334_RS00910	WP_006691631.1	Peptidase U32
*Selenomonas infelix* ATCC 43532	HMPREF9334_RS02810	WP_006691984.1	Peptidase U32
*Selenomonas infelix* ATCC 43532	HMPREF9334_RS06450	WP_006692674.1	Peptidase U32
*Selenomonas noxia* ATCC 43541	HMPREF7545_RS02205	WP_006694041.1	Peptidase U32
*Selenomonas noxia* ATCC 43541	HMPREF7545_RS03310	WP_040571168.1	Peptidase U32
*Selenomonas noxia* ATCC 43541	HMPREF7545_RS04210	WP_006694441.1	Peptidase U32
*Selenomonas ruminantium* subsp. *ruminantium* ATCC 12561	G598_RS0108180	WP_026766556.1	Peptidase U32
*Selenomonas* sp. oral taxon 478	ADJ74_RS10000	WP_050343958.1	Peptidase U32
*Selenomonas* sp. oral taxon 478	ADJ74_RS10520	WP_050344100.1	Peptidase U32
*Selenomonas* sp. oral taxon 478	ADJ74_RS07050	WP_050343039.1	Peptidase U32
*Selenomonas sputigena* ATCC 35185	SELSP_RS05470	WP_006192437.1	Peptidase U32
*Shuttleworthia satelles* DSM 14600	GCWU000342_RS00125	WP_006905076.1	Peptidase U32
*Shuttleworthia satelles* DSM 14600	GCWU000342_RS05060	WP_006906224.1	Collagenase-like protease, PrtC family
*Solobacterium moorei* DSM 22971	H345_RS12725	WP_051240871.1	Collagenase-like protease, PrtC family
*Solobacterium moorei* DSM 22971	H345_RS0101730	WP_028077445.1	Collagenase-like protease, PrtC family
*Streptococcus anginosus* C238 [31]	SANR_RS03650	WP_003035012.1	Peptidase U32
*Streptococcus anginosus* C238 [31]	SANR_RS03655	WP_020999544.1	Collagenase-like protease, PrtC family
*Streptococcus australis* ATCC 700641	HMPREF9421_RS05000	WP_006597381.1	Peptidase U32
*Streptococcus australis* ATCC 700641	HMPREF9421_RS05005	WP_006597406.1	Collagenase-like protease, PrtC family
*Streptococcus constellatus* subsp. *constellatus* SK53	HMPREF1044_RS08695	WP_006270660.1	Collagenase-like protease, PrtC family
*Streptococcus constellatus* subsp. *constellatus* SK53	HMPREF1044_RS08700	WP_037566276.1	Peptidase U32
*Streptococcus constellatus* subsp. *pharyngis* C232 [31]	SCRE_RS05335	WP_006267751.1	Collagenase-like protease, PrtC family
*Streptococcus constellatus* subsp. *pharyngis* C232 [31]	SCRE_RS05340	WP_006267951.1	Peptidase U32
*Streptococcus cristatus* ATCC 51100	HMPREF9422_RS00190	WP_005589468.1	Peptidase U32
*Streptococcus cristatus* ATCC 51100	HMPREF9422_RS04040	WP_005590706.1	Collagenase-like protease, PrtC family
*Streptococcus gordonii* str. Challis substr. CH1 [32]	SGO_RS03645	WP_012000207.1	Peptidase U32
*Streptococcus gordonii* str. Challis substr. CH1 [32]	SGO_RS03650	WP_012000208.1	Collagenase-like protease, PrtC family
*Streptococcus infantis* ATCC 700779	HMPREF9423_RS07500	WP_006148729.1	Peptidase U32
*Streptococcus infantis* ATCC 700779	HMPREF9423_RS07175	WP_006148665.1	Collagenase-like protease, PrtC family
*Streptococcus intermedius* B196 [31]	SIR_RS12835	WP_021002602.1	Collagenase-like protease, PrtC family
*Streptococcus intermedius* B196 [31]	SIR_RS12840	WP_021002603.1	Collagenase-like protease, PrtC family
*Streptococcus mitis* B6 [33]	smi_1316	YP_003446424.1	Collagenase-like protease, PrtC family
*Streptococcus mitis* B6 [33]	smi_0854	YP_003445970.1	Collagenase-like protease, PrtC family
*Streptococcus mutans* UA159 [48]	SMU_759	NP_721176.1	Collagenase-like protease, PrtC family
*Streptococcus mutans* UA159 [48]	SMU_761	NP_721177.1	Collagenase-like protease, PrtC family
*Streptococcus oligofermentans* AS 1.3089 [34]	I872_RS05755	WP_015605207.1	U32 family peptidase
*Streptococcus oligofermentans* AS 1.3089 [34]	I872_RS06980	WP_015605435.1	Peptidase U32
*Streptococcus oralis* Uo5 [35]	SOR_RS05810	WP_000411175.1	Peptidase U32
*Streptococcus oralis* Uo5 [35]	SOR_RS05510	WP_000169101.1	Collagenase-like protease, PrtC family
*Streptococcus parasanguinis* ATCC 15912	HMPREF0833_RS04350	WP_003002878.1	Collagenase-like protease, PrtC family
*Streptococcus parasanguinis* ATCC 15912	HMPREF0833_RS04355	WP_013903889.1	Peptidase U32
*Streptococcus peroris*	HMPREF9180_RS06815	WP_006145781.1	Peptidase U32
*Streptococcus peroris*	HMPREF9180_RS06495	WP_006145710.1	Collagenase-like protease, PrtC family
*Streptococcus salivarius* CCHSS3	SALIVB_RS06560	WP_002886038.1	Peptidase U32
*Streptococcus salivarius* CCHSS3	SALIVB_RS06555	WP_004182776.1	Collagenase-like protease, PrtC family
*Streptococcus sanguinis* SK36 [36]	SSA_1541	YP_001035482.1	U32 family peptidase
*Streptococcus sanguinis* SK36 [36]	SSA_1542	YP_001035483.1	U32 family peptidase
*Streptococcus sobrinus* DSM 20742 = ATCC 33478	BS63_RS0108710	WP_028798546.1	Collagenase-like protease, PrtC family
*Streptococcus sobrinus* DSM 20742 = ATCC 33478	BS63_RS0100290	WP_002962408.1	Collagenase-like protease, PrtC family
*Streptococcus* sp. VT 162	V470_RS05500	WP_044020909.1	Peptidase U32
*Streptococcus* sp. VT 162	V470_RS05200	WP_000169101.1	Collagenase-like protease, PrtC family
*Streptococcus thermophilus* LMG 18311 [37]	STU_RS12905	WP_002952720.1	Peptidase U32
*Streptococcus thermophilus* LMG 18311 [37]	STU_RS12910	WP_002945995.1	Collagenase-like protease, PrtC family
*Streptococcus vestibularis* ATCC 49124	HMPREF9425_RS05965	WP_003097482.1	Peptidase U32
*Streptococcus vestibularis* ATCC 49124	HMPREF9425_RS05960	WP_003094356.1	Collagenase-like protease, PrtC family
*Tannerella forsythia* 92A2	BFO_RS03710	WP_014224272.1	Collagenase-like protease, PrtC family
*Tannerella forsythia* 92A2	BFO_RS05860	WP_014224717.1	Collagenase
*Treponema brennaborense*	TREBR_RS02850	WP_013757721.1	Peptidase U32
*Treponema brennaborense*	TREBR_RS10885	WP_013759230.1	Peptidase U32
*Treponema denticola* ATCC 35405 [38]	TDE0071	NP_970688.1	U32 family peptidase
*Treponema denticola* ATCC 35405 [38]	TDE2262	NP_972862.1	U32 family peptidase
*Treponema putidum* [39]	JO40_RS07200	WP_044978748.1	Peptidase U32
*Treponema putidum* [39]	JO40_RS05960	WP_044978548.1	Peptidase U32
*Treponema vincentii* F0403	HMPREF1222_RS11455	WP_016519531.1	Collagenase-like protease, PrtC family
*Veillonella atypica* KON	HMPREF0870_RS02545	WP_005382667.1	Peptidase U32
*Veillonella atypica* KON	HMPREF0870_RS05775	WP_005376158.1	Peptidase U32
*Veillonella dispar* ATCC 17748	VEIDISOL_RS04770	WP_005386324.1	Peptidase U32
*Veillonella dispar* ATCC 17748	VEIDISOL_RS04185	WP_005386127.1	Peptidase U32
*Veillonella parvula* DSM 2008 [40]	VPAR_RS05935	WP_012864557.1	Peptidase U32
*Veillonella parvula* DSM 2008 [40]	VPAR_RS05390	WP_012864475.1	Peptidase U32

The microorganism associated with each gene annotation is indicated in the first column along with the corresponding reference when available. Taxonomical and protein assignments were identified in the metatranscriptome analysis of root biofilms.

**Figure 1.**
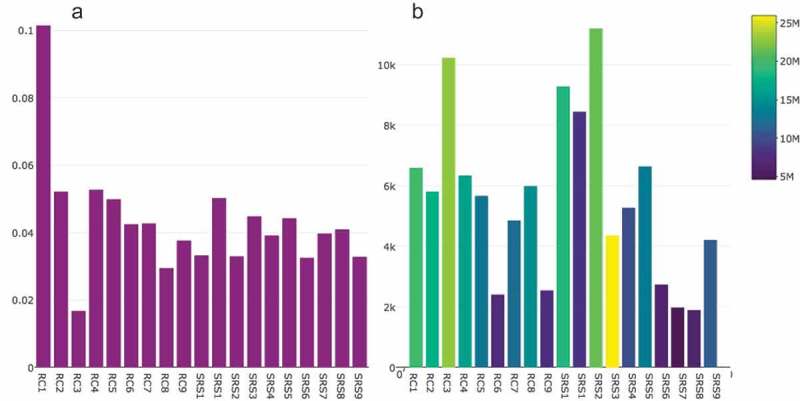
Bacterial collagenolytic proteases present in samples from sound root surfaces (SRS) and root caries (RC). (a) Proportion (%) of bacterial collagenolytic proteases based on the total read count per sample; (b) Number of reads mapped to bacterial collagenolytic protease genes (yellow = sample with more total reads per sample; blue = sample with less total reads per sample).

Overall, bacterial collagenolytic proteases showed low levels of expression. The higher proportion of reads assigned to the bacterial collagenolytic proteases was 0.1% of total reads (RC_7). The other samples had an average of proportion of reads assigned to the bacterial collagenolytic proteases of 0.04% for SRS and 0.05% for the RC group, and no statistically significant differences were found (*t* test; *p* = 0.2) (Figure 1(a,b)). However, the number of collagenase genes with no expression (number of reads = 0) was SRS = 73.1 ± 9.6 (36.4%) and RC = 109.1 ± 23.7 (54.3%) (*t* test; *p* = 0.000). Thus, in spite of similar number of reads in RC and SRS, the number of genes encoding collagenases in RC was lower than in SRS.

**Figure 2.**
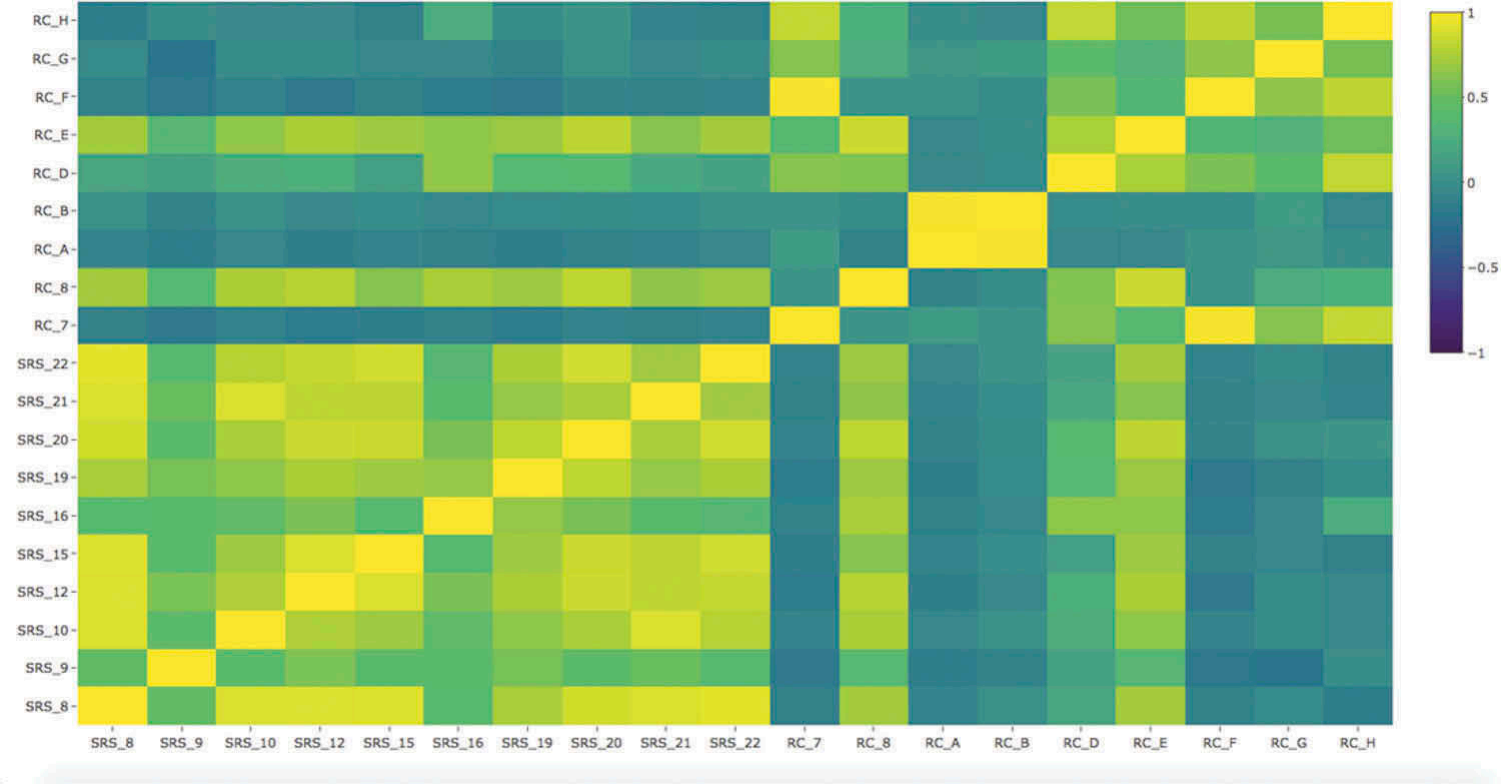
Heatmap showing the distances between the samples as calculated from the normalized count data of the gene expression of bacterial collagenolytic proteases. RC = root caries samples; SRS = sound root surfaces samples.

The heatmap showing the distances between the samples (*n* = 19) is represented in Figure 2. It takes into account the level of expression of the genes that code bacterial collagenolytic proteases within the sample for each group. There was less sample-to-sample variation between the SRS samples than the RC samples (RC_8, RC_D and RC_E differ from the other RC samples). The diversity of gene expression patterns in the RC samples could represent differences in the lesion characteristics, such as caries stages and lesion sizes.

**Figure 3.**
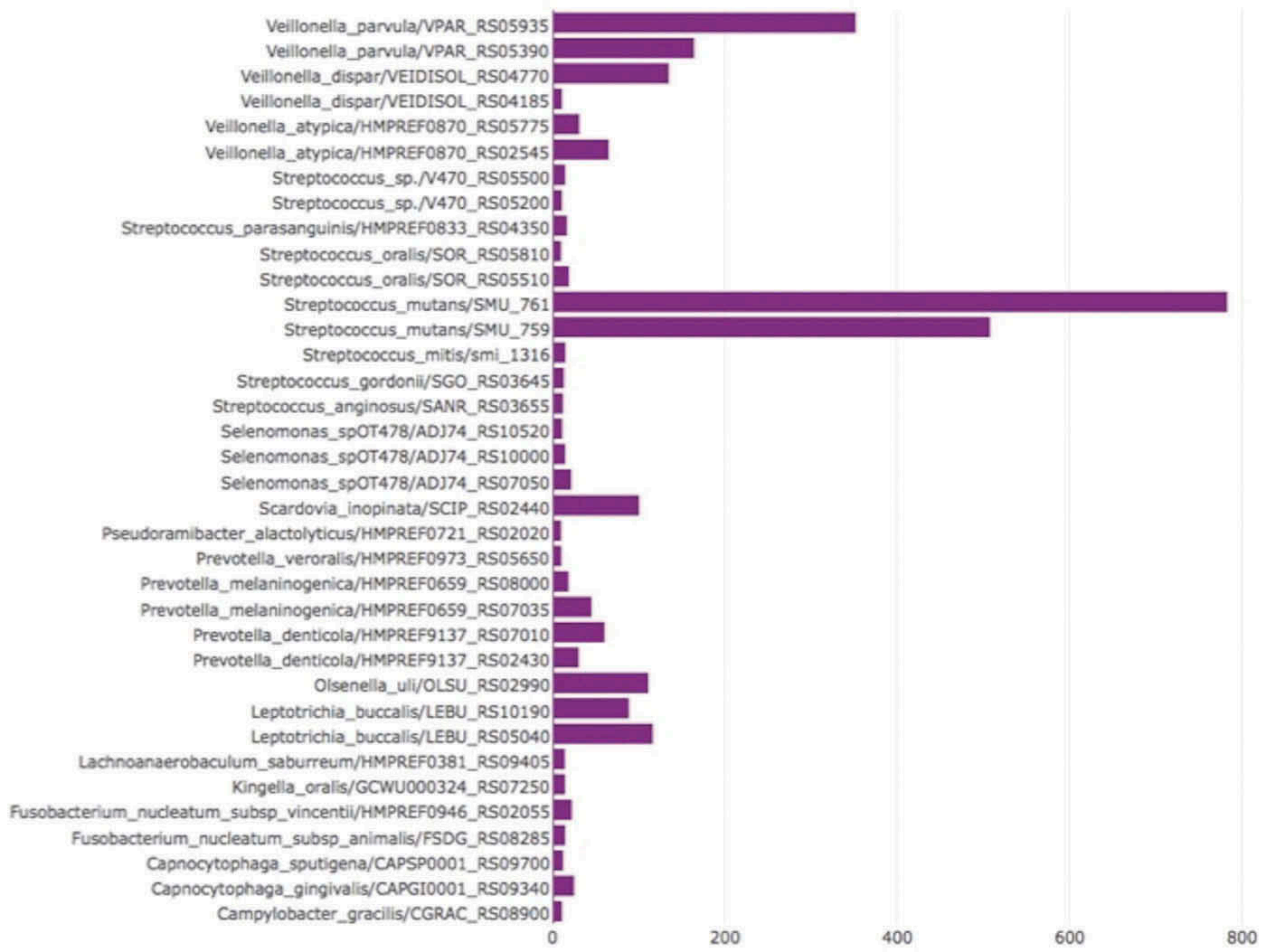
Gene expression level (median of expression value) of putative bacterial collagenolytic proteases (presented as ‘bacterial species name/gene locus tag’) in root caries. Only genes that had gene expression level >10 are displayed.

Figure 3 shows the median expression value of collagenolytic proteases in RC lesions, i.e. the median of the normalized read numbers. Eight collagenolytic proteases had a median of expression value higher than 100, including those from *S. mutans, Veillonella parvula, V. dispar, Leptotrichia buccalis, Olsenella uli*, and *Scardovia inopinata*. It is important to point out that in two RC samples, *S. inopinata* had the highest collagenolytic protease expression value (RC_A = 14,838.83 and RC_B = 3,305.65), although the median was lower than other species. Three collagenolytic proteases had expression values higher than 200, meaning that these were very highly expressed in RC: SMU_761 and SMU_759 from *S. mutans* and RS05935 from *V. parvula. S. mutans* possessed collagenolytic proteases with the highest gene expression in RC, while *L. buccalis* possessed collagenolytic proteases with the highest gene expression in SRS.

**Figure 4.**
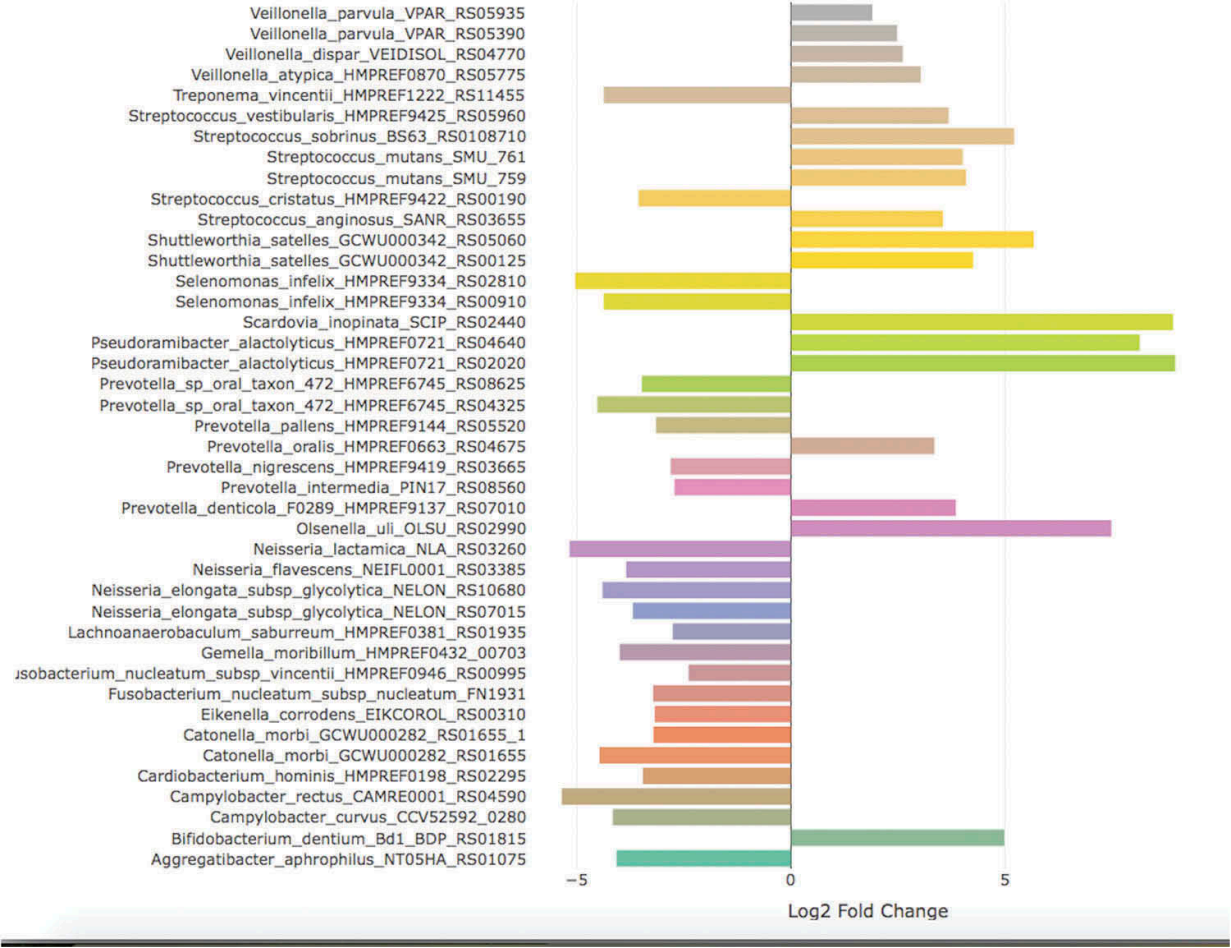
Genes with significant differential expression coding for bacterial collagenolytic proteases (presented as ‘bacterial species name/gene locus tag’) in the metatranscriptome analysis of root biofilms. Positive log2FoldChange means up-regulated genes in root caries, while negative log2FoldChange means up-regulated in sound root surfaces.

Using a very high cut-off (FDR <10^−^^3^) for considering differential expression between sound and carious biofilms, we observed 42 bacterial collagenolytic proteases with significant differential expression: 24 were overexpressed in SRS and 18 in RC (Figure 4). *P*. *alactolyticus* [HMPREF0721_RS02020], *S. inopinata* JCM 12537 [SCIP_RS02440], *P. alactolyticus* [HMPREF0721_RS04640], and *O. uli* DSM7084 [OLSU_RS02990] were the organisms with highly overexpressed proteases in RC (Log2FC>8), but no expression in SRS.

## Discussion

The current understanding of the microbial functions in RC and dentine caries remains limited compared with enamel caries. In a recent review of caries ecological hypotheses, it was proposed that bacteria play a role in the degradation of the organic components of teeth [3]. Although a lot of bacteria are found to secrete collagenolytic proteases, their roles and the mechanisms involved in cariogenic processes are still largely unknown [8]. This is the first study showing bacterial collagenolytic proteases gene expression within the metatranscriptome of clinical dental biofilms with and without RC. Our findings show that a few species were responsible for high expression of genes that code for bacterial collagenolytic proteases in RC, namely *S. mutans, V. parvula, V. dispar*, and *S. inopinata*.

The progression of caries lesions involves the degradation of the collagen matrix in the root hard tissues. The collagen protein family is characterized by the presence of the proline-rich tripeptide ‘Gly-X-Y’, forming a triple helix of polypeptide chains in which the glycine residue is positioned in the centre [41]. Collagen type I, the most common in dentine, has a heterotrimer structure. The collagen structure contributes to the molecular stabilization and mechanical properties of dentine. Only a specific group of proteases, the collagenases, are able to degrade collagen. The triple-helix is interrupted in its internal structure by digesting the triple-helix three-quarters of the way from the terminal amino group ‘Gly-Leu’ bond. This may cause intramolecular flexibility and allow specific proteolytic cleavage [41]. Bacterial collagenolytic proteases include some metalloproteases of the M9 family and some serine proteases. These are distributed in the S1, S8, and S53 families and also some members of the U32 family, mainly from pathogenic bacteria [8]. In this study, protease PrtC was detected to have a relatively low gene expression levels. Other protease families were not detected in the genomes annotation, and these still remain to be investigated (i.e. the M9, S8, and S53 families).

Dental caries occurs not by continuous demineralization but by alternating demineralization and remineralization. According to a recent theory proposed by Takahashi and Nyvad (2016), the exposed collagen is broken down and the collagen content may be denatured during a second stage of RC. The theory suggests that collagen matrix degradation could only be possible after demineralization because the substrate is not accessible by collagenases in the mineralized tissue. Some endogenous collagenases have been shown to be involved in this process [9,10,42]. MMPs, zinc-dependent endopeptidases, are able to cleave denatured collagen. They function in tissue development and repair and in pathological processes as well [43]. It has been found that bacterial collagenases have no activity during demineralization in an acid environment (pH 4.3) [43,44], and it was shown that collagenase works during the remineralizing phase and predominantly attacks the organic matrix of the root after demineralization [44]. However, collagen degradation products are known to be released from dentine when treated with lactic acid and bacterial collagenase or trypsin [45]. Therefore, acids from bacterial metabolism may render dentinal collagen more susceptible to host and microbial proteases such as those of the U32 family.

It has been reported that *S. mutans* is not associated with collagen matrix degradation in cavitated RC [46,47]. However, in this study, we detected high expression of genes SMU_761 and SMU_759 (*S. mutans* UA159). Both genes encode collagenase-like protease, PrtC family (peptidase U32 family) [48]. SMU_761 codes for a 428 aa protein, while SMU_759 encodes a 308 aa protein. *S. mutans* is widely known as an important aetiological agent of dental caries, due to its involvement in biofilm formation and its aciduricity and acidogenicity. Furthermore, most culture-based studies have shown a strong relationship between RC and these bacteria, which have higher isolation frequencies and/or higher proportions on carious root surfaces [49–53]. Our results suggest that the collagenase activity could also be an important virulence factor of *S. mutans* in RC. These proteases were also elevated under conditions of glucose excess in another *in vitro* transcriptome study [54].

Along with *S. mutans*, two species of *Veillonella* (*V. parvula* and *V. dispar*) showed high collagenase gene expression levels in RC. These species have been implicated in dentinal caries due to their overexpressed functions in caries lesions, inferring a role in disease [18]. Other species such as *P. alactolyticus, S. inopinata*, and *O. uli* had high differential expression in RC when compared to SRS. These species have been included in the complex microbial community of coronal caries [15] and RC [53,55–58], but their roles and functions have been underexplored.

A higher level of gene expression of some bacterial collagenases was observed in samples from the control group of this study (supragingival biofilm – SRS). Periodontopathogens, such as *Prevotella intermedia*, showed high differential expression in SRS. The SRS group included patients in preventive periodic maintenance for periodontal disease: the U32 proteases explored here have been previously related to periodontal disease [16]. So this result could be linked to collagen degradation of periodontal tissues.

It is important to acknowledge that we cannot state that there is activity of bacterial collagenolytic proteases in the degradation of dentine because our data are based on gene expression and the enzymes could be inactive *in vivo*. It is also important to note that other organisms not included as reference genomes in this analysis could be expressing collagenases, as the analysis presented here relies on the current reference databases and other not yet identified collagenases (for example, those currently identified as hypothetical proteins) may play an important role in collagen degradation. This work represents a preliminary screening of transcripts coding for collagenases using clinical data and the validation is being planned in further investigations. However, it is important to point out that the level of protease transcripts observed in this study may indicate the importance of this function within the RC biofilm communities, considering that the transcription of irrelevant genes would be a waste of energy to the microorganisms.

The results suggest that the U32 proteases could be related to RC lesions (carious dentine). The contribution of some species in dentine degradation should be further investigated, such as *S. mutans, V. parvula*, and *V. dispar* (high gene expression level in RC), as well as *P. alactolyticus, S. inopinata*, and *O. uli* (high differential expression in RC when compared to SRS). Our results provide novel insights into the collagenase activity of some bacterial species in RC. These studies lay the foundations for further investigations involving the use of proteomic tools, to better understand the aetiology of RC, and microbial metabolic activities leading to disease progression. These proteases may have potential for future biotechnological and medical applications serving as targets for the development of therapeutic agents.
